# The Prevalence of Hypertension Accompanied by High Homocysteine and its Risk Factors in a Rural Population: A Cross-Sectional Study from Northeast China

**DOI:** 10.3390/ijerph14040376

**Published:** 2017-04-03

**Authors:** Ye Chang, Yuan Li, Xiaofan Guo, Yintao Chen, Dongxue Dai, Yingxian Sun

**Affiliations:** Department of Cardiology, the First Hospital of China Medical University, Shenyang 110001, China; chang.ye@stu.xjtu.edu.cn (Y.C.); xi.aohan1989@163.com (Y.L.); guoxiaofan1986@foxmail.com (X.G.); chenyintao1990@126.com (Y.C.); 18240409506@163.com (D.D.)

**Keywords:** hypertension, homocysteine, rural population, cross-sectional study

## Abstract

Previous studies found that hypertension and high homocysteine (HHcy) exhibited a synergistic effect on the risk of cardiovascular diseases. This study aimed to investigate the prevalence of hypertension accompanied by HHcy and its risk factors in the rural areas of northeast China. This study was conducted using a stratified cluster random sampling method, and included 6529 subjects with complete data. Demographic characteristics were obtained from a questionnaire. Blood pressure and anthropometric indices were measured, and serum indices were analyzed. Hypertension accompanied by HHcy was defined as hypertension plus HHcy [homocysteine (Hcy) > 10 µmol/L]. The mean concentration of Hcy was 17.29 μmol/L in the general population. The prevalence of hypertension accompanied by HHcy was so high that it reached 45.1% of our study population and accounted for 86.8% of the total participants with hypertension. Multiple logistic regression analysis indicated that the modifiable risk factors of hypertension accompanied by HHcy included obesity, diabetes, dyslipidemia, and inactive physical activities. We found that the mean level of Hcy, and the prevalences of HHcy and hypertension accompanied by HHcy were very high among the rural population of northeast China. Obesity, diabetes, dyslipidemia, and inactive physical activities were modifiable risk factors of hypertension accompanied by HHcy.

## 1. Introduction

Hypertension has been identified as an independent risk factor of cardiovascular diseases (CVD) and is the number one cause of deaths all over the world [1]. Hypertension is becoming a global epidemic in both children and adults, in both the rural population and urban population [2,3]. In 2015, the National Center for Cardiovascular Diseases (NCCD) in China published the Report on Cardiovascular Diseases in China (2015) [4]. The Report indicated that approximately one in five Chinese (about 270 million) individuals suffered from hypertension and that mortality due to hypertension accounted for 2.04 million cases, representing 24.6% of the total number of deaths. According to the Reports on Nutrition and Chronic Disease Status of Chinese Residents (2015) [5], the prevalence of hypertension was 25.2% in adults aged ≥ 18 years. Our previous study based on subjects ≥ 35 years found that the prevalence of hypertension was so high that it reached 51.1% in the rural population of northeast China [3]. It was worth noting that about 75% of hypertensive patients also exhibited high homocysteine (HHcy) in China [6].

Homocysteine (Hcy) is an intermediate product of methionine metabolism, and its concentration in fasting plasma is maintained by homeostasis (5–15 μmol/L) under physiological conditions [7]. In 2006, HHcy was defined as a Hcy concentration >10 µmol/L by the American Society of Hypertension (ASH) and the American Stroke Association (ASA) [8]. Several lines of evidence suggest that HHcy is an independent risk factor for CVD [9,10]. Previous studies found that hypertension and HHcy exhibited a synergistic effect on the risk of CVD [11,12]. In 2008, hypertension accompanied by HHcy (H-type hypertension) was proposed, which meant hypertension plus HHcy (Hcy > 10 µmol/L) [6]. In the study of the China Stroke Primary Prevention Trial (CSPPT), which was a randomized, double-blind clinical trial including 20,702 adults with hypertension, hypertension accompanied by HHcy accounted for 79.7% of the untreated hypertensive patients [13]. In another study conducted on the nomadic minority residing in China, the prevalence of hypertension was 35.1% in Kazakh individuals, and the percentage of hypertension accompanied by HHcy accounted for 87.6%; the prevalence of hypertension was 30.6% in Uyghur individuals, and the percentage of hypertension accompanied by HHcy accounted for 88.0% [14]. In a study based on hospitalized patients during the acute phase of a stroke, the percentage of hypertension accompanied by HHcy reached 90.4% of hypertensive patients [15]. One retrospective study from China including 13,192 hospital-based patients found that the prevalence of hypertension was 36.1%, and the percentage of hypertension accompanied by HHcy accounted for 74.4% [16]. However, data on cases of hypertension accompanied by HHcy in the rural population of northeast China are rare.

The Liaoning province is located in northeast China, where the winter is long and cold. In rural areas, the overall economic and education levels are low. Most of the residents are farmers and are engaged in heavy manual agricultural labor. The farmers still lead a traditional lifestyle, such as working from sunrise to sundown, growing vegetables and rice to support a self-sufficient life [17]. They have similar socioeconomic backgrounds and dietary habits because they rarely move and migration accounts for only a small proportion of the population. During the cold winter from November to April in the second year of the study, there was a lack of fresh vegetables and they mainly ate meat, tofu, and pickles, such as Chinese sauerkraut. There is a lack of serious study analyzing local public health needs.

Therefore, our study aimed to investigate the prevalence of hypertension accompanied by HHcy and explore its risk factors in a rural population of northeast China. It may be useful for providing proper guidelines for CVD prevention programs.

## 2. Materials and Methods

### 2.1. Study Population

From July 2012 to August 2013, we conducted this cross-sectional study in rural areas of the Liaoning province (located in northeast China), using a multistage, stratified, random cluster sampling scheme. A representative sample of individuals was chosen to present the prevalence, incidence, and natural history of cardiovascular risk factors. In the first step, three counties (i.e., Dawa, Zhangwu, and Liaoyang County) were chosen randomly, to represent the eastern, southern, and northern regions of the Liaoning province, respectively; in the second step, one town was randomly chosen from each of the three counties; in the third step, eight to 10 rural villages were randomly chosen from each town and a total of 26 rural villages were finally chosen. To take part in the study, participants had to be at least 35 years or older and needed to permanently reside in the area. Furthermore, participants who were pregnant, or who were suffering from a malignant tumor or mental disorders, were not included in this study. A total of 14,016 eligible participants from the 26 chosen villages were invited to participate and 11,956 participants agreed to attend and completed the study, with a response rate of 85.3%. The methods were consistent with our previous studies [17,18]. Blood samples were collected and were frozen at −20 °C, which were tested in a central laboratory. A routine blood examination and serum analysis were conducted, according to the study protocol. In the last phase of the study, we recorded the concentration of Hcy; however, only half of the Hcy levels could be measured due to a lack of research funding. Therefore, we selected the blood samples from four to five rural villages in each county at random and the concentration of Hcy in these clusters was detected. The final sample consisted of 6529 participants (2990 males and 3539 females), with non-missing homocysteine levels in the present study.

### 2.2. Ethical Statement

All participants provided written informed consent after having been informed of the objectives, benefits, medical items, and confidentiality of personal information. If the participants were illiterate, their proxies would sign the written informed consent. The study was approved by the Ethics Committee of the China Medical University (Shenyang, China, ethical approved project identification code: 2011-2-2) and all of the procedures were performed in accordance with ethical standards.

### 2.3. Data Collection

A detailed description of the methods can be found in our previous studies [17,18]. Our investigation was conducted by experienced cardiologists and trained nurses via a single visit. One clinic was selected in each village, where the participants were interviewed using a standardized questionnaire. The standardized questionnaire was designed by statistical experts and clinical specialists, and included questions on demographic characteristics, lifestyle risk factors, dietary habits, family income, hypertensive medication use, and other variables. Before conducting the survey, we asked all eligible investigators to undergo organized training on the study purpose and procedures, how to administer the questionnaire, standard methods of measurement, and the importance of standardization. At the end of the training period, a strict test was performed and only those who scored perfectly became investigators. During data collection, our study inspectors provided further instruction and support, as needed. All of the questionnaires were administered by the quality control committee and the unqualified questionnaires were reinvestigated.

The participants were classified by marital status into two groups: married or living with partner; and unmarried, divorced, or widowed. The participants were classified by ethnicity into two groups: Han or others (including ethnic minorities in China, such as Mongol and Manchu). The participants were classified by family income into three groups: 5000 or less, 5000–20,000, and 20,000 or over, China Yuan (CNY)/year. The participants were classified by educational level into three groups: (1) low level was defined as no schooling, incomplete primary education, or primary education; (2) middle level was defined as three to four years of secondary education; and (3) high level was defined as college and university education. Alcohol consumption was also calculated using the questionnaire. The participants were asked to answer the following questions: (1) Do you drink regularly?; If yes, (2) what kind do you drink: beer, red wine, or hard liquor?; (3) How many times do you drink per week?; and (4) How much do you drink per time? The amount of pure alcohol was calculated according to the frequency and amount of drinking. In China, the ethanol weight content differed among the beverages as follows: 5% in beer, 12.5% in red wine, and 45% in hard liquor [19]. One drink was equivalent to a mean consumption of 15 g ethanol [19,20]. According to the level of alcohol consumption per day, the participants were classified into three groups: nondrinkers (abstainers, or no history of alcohol consumption); moderate drinkers (≤1 drink per day for women and ≤2 drinks per day for men); and heavy drinkers (>1 drink per day for women and >2 drinks per day for men) [21]. The definition of an alcohol drinking habit was described in detail in our previous study [19]. In the present study, subjects with an alcohol drinking habit included moderate drinkers and heavy drinkers.

In 2010, the American Heart Association (AHA) proposed a new concept, “ideal cardiovascular health” (CVH), which consisted of seven CVH metrics: body mass index (BMI), physical activity, diet score, smoking, fasting plasma glucose (FPG), total cholesterol (TC), and blood pressure (BP). The seven CVH metrics were further classified into ideal, intermediate, and poor status. Therefore, in the present study, we used these criteria to define the following variables, as our previous studies described [17,18].

### 2.4. Smoking Status

Smoking status was accessed by the following questions [17,18]: (1) Have you been a smoker?; If yes, (2) do you smoke now?; (3) Have you ever quitted smoking?; and If yes, (4) how long ago did you quit smoking? Based on these questions, smoking was classified into three groups: ideal (never smoker or quitting smoking >1 year), intermediate (quitting smoking ≤ 1 year), or poor (current smoker).

### 2.5. Diet Score

The questionnaire included questions about the average consumption (grams per week) of several food items, which consisted of legumes, vegetables, fruits, fish, poultry, and salt intake. A diet score was calculated using the following five components: (1) legumes and cereals as basic food; (2) ≥500 g fruits and vegetables per day; (3) ≤100 g red meat per day; (4) regular (in most weeks) intake of soybean products and/or unprocessed fish; and (5) preference for non-salty food, according to the current “Dietary Guidelines for Chinese Residents” [22].

### 2.6. Physical Activity

We initially wanted to assess physical activity according to the following questions, which were consistent with the AHA’s criteria: (1) Do you regularly exercise?; If yes, (2) how many times do you exercise per week and how long do you exercise for each time?; and (3) What is your most commonly pursued mode of exercise? 1 = walking, 2 = running, 3 = swimming, 4 = ball games, 5 = mountaineering, 6 = others. However, during the visit, we found that these questions didn’t apply to the rural populations because they didn't actively take exercise such as walking, running, swimming, ball games, or mountaineering. Most of the populations in the rural areas of the Liaoning province were farmers who were too exhausted to engage in the agricultural work during the spring, summer, and autumn. In the winter, the temperature in this area would always be below −20 °C. Therefore, they preferred to watch television, and play mahjong or poker in their leisure time. Considering this, we decided to adopt another method, described elsewhere, to measure occupational physical activity [23]. Briefly, participants were asked the question: “which category do you think your occupational physical activity belongs to?”. Occupational physical activity was classified into three categories: (1) low was defined as participants who reported light levels of occupational physical activity, such as the elderly, crippled, and those with paralysis; (2) moderate was defined as participants who reported moderate occupational physical activity, such as a driver or office worker; and (3) high was defined as participants who reported a high level of occupational physical activity, such as manual agricultural activities and those who were miners. To be consistent with the AHA’s criteria [24], the low, moderate, and high levels of occupational physical activity were regarded as equivalents to the poor, intermediate, and ideal status of physical activity in our studies [17,18].

### 2.7. Category of Blood Pressure

Consistent with the AHA’s protocols [25], the study participants were asked to avoid caffeinated beverages or exercise for at least 30 min, and rested in a sitting position for at least 10 min before the measurement. The systolic blood pressure (SBP, mmHg) and diastolic blood pressure (DBP, mmHg) were measured using a standardized automatic electronic sphygmomanometer (HEM-907; Omron, Japan). During the measurement, the participants were seated with the arm supported at the level of the heart. The BP was measured three times at 2-min intervals after at least 5 min of rest, and the mean was calculated. Consistent with the AHA’s definition [24], BP was classified into three groups: ideal (SBP < 120 mmHg and DBP < 80 mmHg, untreated), intermediate (SBP 120–139 mmHg or DBP 80–89 mmHg, or treated to goal), and poor (SBP ≥ 140 mmHg or DBP ≥ 90 mmHg). Hypertensive medication use was assessed using the questionnaire, by the following questions: (1) In the last two weeks, was there any day on which you did not take your antihypertensive drugs? 1 = yes, 2 = no; (2) Do you take antihypertensive drugs by following the doctor's advice? 1 = regular (≥9 months/year), 2 = discontinuous (3–9 months/year), 3 = occasional (<3 months/year), 4 = no; (3) What is the antihypertensive efficacy after you take antihypertensive drugs? 1 = ideal (treated to goal for more than 9 months/year), 2 = intermediate (treated to goal for 6–9 months/year), 3 = poor (treated to goal for less than 6 months/year), 4 = I don’t know because I don’t measure BP; (4) Have you sometimes forgotten to take antihypertensive drugs in the past year? 1 = yes, 2 = no; (5) When you travel or leave the house, do you sometimes forget to take your medication? 1 = yes, 2 = no; (6) When you feel that your BP is controlled, do you sometimes stop taking your medication? 1 = yes, 2 = no; (7) Have you ever stopped taking antihypertensive drugs or decreased the dose without first warning your doctor because you felt worse when you took them? 1 = yes, 2 = no; (8) Have you stopped taking antihypertensive drugs because you were concerned about the side effects? 1 = yes, 2 = no; (9) How often do you have difficulty in remembering to take antihypertensive drugs? 1 = never, 2 = almost never, 3 = sometimes, 4 = frequently, 5 = always; (10) Questions about antihypertensive drugs including the number of drugs used, the name of the drugs, the dose (bottles/year and pills/frequency), and the frequency (times/day).

### 2.8. Category of BMI

The participants were asked to wear light clothing and no shoes. Their weight was measured to 0.1 kg and their height was measured to 0.1 cm, respectively. The participants were asked to stand at the end of normal expiration. Their waist circumference (WC) was measured to 0.1 cm, using a non-elastic tape at the umbilicus. Their BMI was calculated using the following formula: BMI = weight/height (kg/m^2^). The participants were classified by BMI into three groups: normal (BMI < 22.9 kg/m^2^), overweight (23 ≤ BMI < 27.4 kg/m^2^), and obese (BMI ≥ 27.5 kg/m^2^), according to the obesity criteria for Asian people recommended by the World Health Organization (WHO) [26].

### 2.9. Serum Analysis

All of the participants were asked to fast for 12 h before the early-morning blood samples were collected. Blood samples were obtained from an antecubital vein and were collected in vacutainer tubes containing EDTA. Blood samples were centrifuged immediately to isolate the serum, which was frozen at −20 °C and carried to a central laboratory for testing. Blinded duplicate samples were analyzed using an Olympus AU640 auto analyzer (Olympus, Kobe, Japan). Serum concentrations of FPG, TC, triglyceride (TG), low density lipoprotein (LDL), high density lipoprotein (HDL), uric acid (UA), creatinine (Cr), blood urea nitrogen (BuN), and other routine blood biochemical indices were analyzed. TC was classified into three groups: ideal (<200 mg/dL (5.18 mmol/L), untreated), intermediate (200–239 mg/dL (5.18–6.21 mmol/L) or drug treated to goal), or poor (≥240 mg/dL (6.21 mmol/L)) [24]. FPG was classified into three groups: ideal (<100 mg/dL (5.6 mmol/L), untreated), intermediate (100–125 mg/dL (5.6–7.0 mmol/L) or drug treated to goal), or poor (≥126 mg/dL (7.0 mmol/L)) [24]. 

### 2.10. Estimated Glomerular Filtration Rate

The glomerular filtration rate (GFR) was estimated using the equation originating from the Chronic Kidney Disease Epidemiology Collaboration (CKD-EPI) equation [27].

### 2.11. Simple Hypertension, HHcy and Hypertension Accompanied by HHcy

Plasma Hcy was measured by an enzyme cycling method using a Hitachi 7020 Q3 Automatic Analyzer (Hitachi, Japan). HHcy was defined as the concentration of Hcy ≥ 10 μmol/L [8]. Simple hypertension was defined as participants with hypertension and normal Hcy. H-type hypertension was defined as participants with hypertension and HHcy [6,14]. However, we do not believe that there is a pathological entity known as H-type hypertension. So, in this study, we replaced H-type hypertension with the term hypertension accompanied by HHcy.

### 2.12. Statistical Analyses

Descriptive statistics were calculated for all of the variables. Continuous variables were reported as mean ± standard deviation (SD), and the differences in the subgroups were compared using one-way analysis of variance (ANOVA). Categorical variables were reported as numbers and percentages, and the differences in the subgroups were performed using the χ^2^ test. Multivariable logistic regression analyses were used to access the association between potential risk factors and the prevalence of hypertension accompanied by HHcy, which were adjusted for age (or gender), race, marital status, education, family income, smoking, drinking, physical activity, diet score, BMI, WC, TC, TG, LDL, HDL, eGFR, UA, Cr, and BuN. We also addressed multicollinearity by centering the covariant variables. The variance inflation factor (VIF) scores, which varied from 1.06 to 1.96, suggested that multicollinearity was not a substantive problem in the data, thus allowing for a meaningful interpretation of the results. Analyses were presented as odds ratios (ORs) and corresponded to 95% confidence intervals (CIs). All of the statistical analyses were performed using SPSS version 22.0 software (SPSS Inc., Chicago, IL, USA), and *p* < 0.05 was considered to be statistically significant.

## 3. Results

This study included a total of 6529 subjects (2990 men and 3539 women) with complete data. The subjects were classified into four groups based on their BP status and Hcy concentration (i.e., control group, simple hypertension group, simple HHcy group, and hypertension accompanied by HHcy group). There were 449 subjects suffering from simple hypertension, 2545 subjects suffering from simple HHcy, and 2943 subjects suffering from hypertension accompanied by HHcy, accounting for 6.9%, 39.0%, and 45.1% of our study population, respectively. The subjects suffering from hypertension accompanied by HHcy accounted for 86.8% of the total participants with hypertension.

Table 1 shows the general characteristics of the four groups. Compared to the control group, the subjects in the other three groups were older (*p* < 0.001). The values of SBP, DBP, BMI, WC, TC, TG, LDL, FPG, Cr, BuN, UA, and Hcy differed significantly among the four groups (all *p* < 0.001), and participants with hypertension accompanied by HHcy had higher values. The values of the estimated glomerular filtration rate (eGFR) and HDL showed the opposite trend (both *p* < 0.001). Participants with hypertension accompanied by HHcy tended to be male, a smoker, drinker, and of a Han race. Furthermore, participants with hypertension accompanied by HHcy had a lower family income, lower education level, and participated in less physical activity compared to participants without hypertension and HHcy.

Table 2 shows the distribution of six CVH metrics according to BP status and Hcy concentrations. There were no differences in the diet score among the four groups. Besides the diet score, the distribution of the other five CVH metrics showed significant differences. Overall, subjects with hypertension accompanied by HHcy had lower percentages of ideal CVH metrics (all *p* < 0.05). In the hypertension accompanied by HHcy group, the ideal smoking level was the most prevalent ideal CVH metric (60.4%); more than half of the subjects had ideal TC (50.2%); the prevalence of the other ideal CVH metrics was 46.4% for FPG, 42.2% for physical activity, 26.0% for BMI, and 10.8% for diet score.

**Figure 1:**
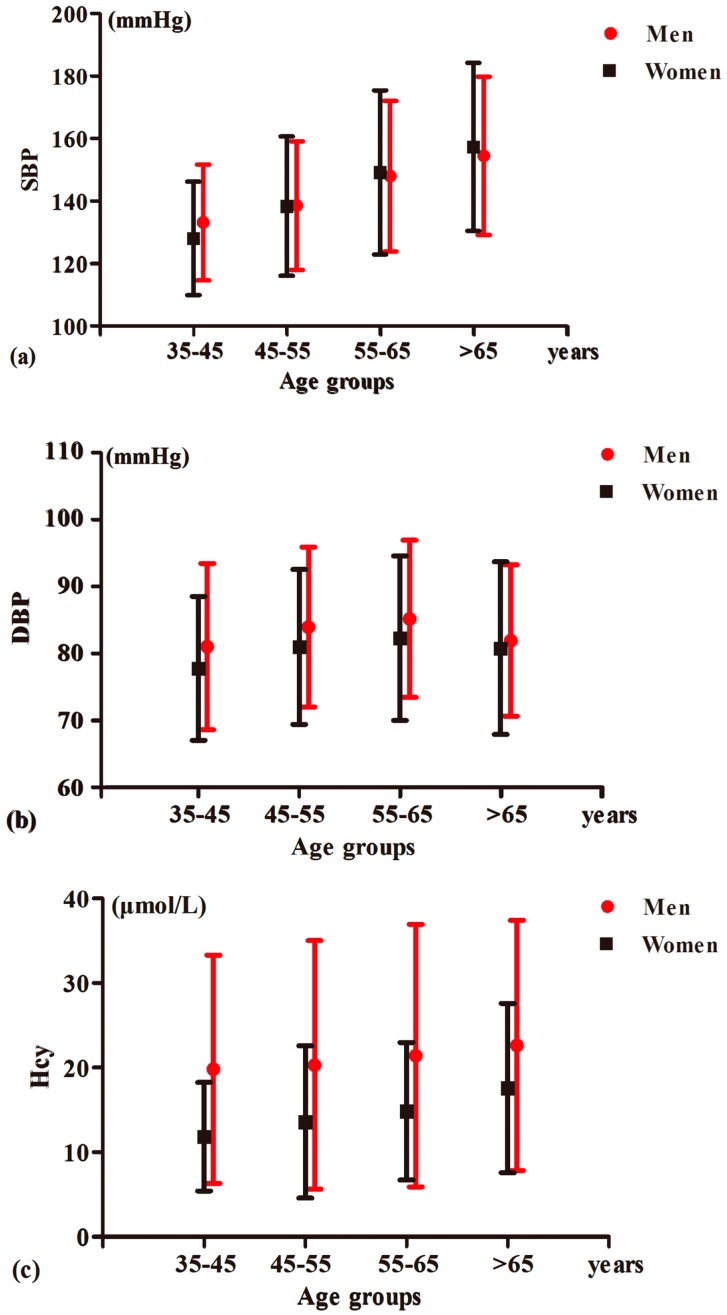
The association between SBP (**a**); DBP (**b**); and Hcy (**c**), with age in both genders. The values of SBP, DBP, and Hcy are expressed as mean ± SD. DBP: diastolic blood pressure; Hcy: homocysteine; SBP: systolic blood pressure; SD: standard deviation.

Subjects were divided into four age groups: 35–45, 45–55, 55–65, and >65 years. In each age group, subjects were further classified into two groups by gender. As shown in Figure 1, the mean values of SBP (a), DBP (b), and Hcy (c) increased with age. In the same age group, women tended to have lower values of SBP (a), DBP (b), and Hcy (c) compared to men.

**Figure 2:**
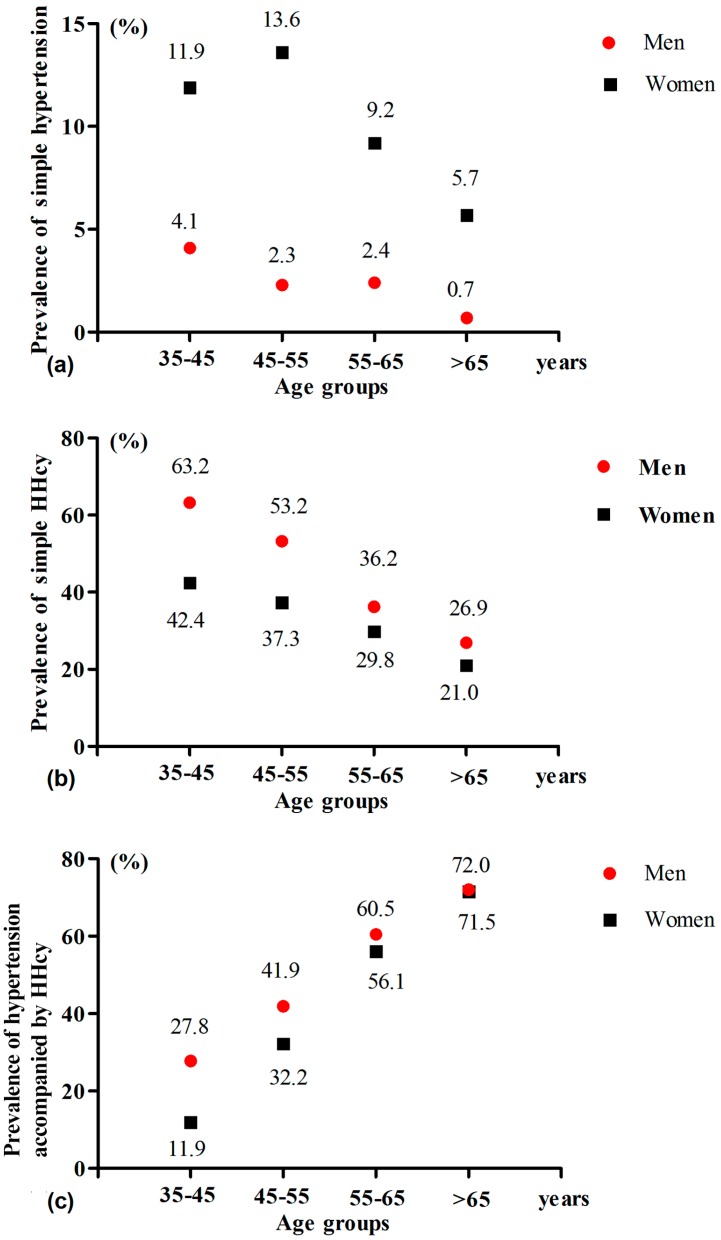
The prevalence of simple hypertension (**a**) simple HHcy (**b**) and hypertension accompanied by HHcy (**c**) in four age groups classified by gender. HHcy: high homocysteine.

Subjects were divided into four age groups: 35–45, 45–55, 55–65, and >65 years. As shown in Figure 2a,b, the prevalence of simple hypertension and simple HHcy exhibited an inverse relationship with age. It is well known that the prevalence of hypertension (including simple hypertension and hypertension accompanied by HHcy) increases with age. In the present study, the prevalence of simple hypertension decreased with age, suggesting that the prevalence of hypertension accompanied by HHcy dramatically increased with age. In other words, older participants with hypertension also often had HHcy. In the same age group, women tended to suffer more from simple hypertension and less simple HHcy compared to men. As shown in Figure 2c, the prevalence of hypertension accompanied by HHcy increased with age. In men, the prevalence of hypertension accompanied by HHcy was 27.8%, 41.9%, 60.5%, and 72.0% among the four age groups, respectively; in women, the prevalence was 11.9%, 32.2%, 56.1%, and 71.5% among the four age groups, respectively. It was interesting that the prevalence of hypertension accompanied by HHcy in women was significantly lower than that in men when they were less than 55 years old. However, this difference disappeared when they were more than 55 years old. These results suggest that the estrogenic hormone may play a protective role against hypertension accompanied by HHcy.

Multivariable logistic regression analyses were conducted to assess the associations between modifiable risk factors and hypertension accompanied by HHcy, after having adjusted for age, race, marital status, education, family income, smoking, drinking, physical activity, diet score, BMI, WC, TC, TG, LDL, HDL, eGFR, UA, Cr, and BuN. Table 3 shows the ORs and 95% CIs of hypertension accompanied by HHcy in both genders. TG (OR = 1.12; 95% CI: 1.06–1.19 in men; OR = 1.09; 95% CI: 1.02–1.16 in women) and LDL (OR = 1.60; 95% CI: 1.34–1.90 in men; OR = 1.69; 95% CI: 1.43–1.99 in women) were both independent risk factors for hypertension accompanied by HHcy. According to the AHA’s criteria, TC, BMI, and FPG were classified into three groups: ideal, intermediate, and poor. Subjects in the poor status of BMI (OR = 3.41; 95% CI: 2.63–4.41 in men; OR = 3.12; 95% CI: 2.45–3.96 in women), TC (OR = 1.88; 95% CI: 1.24–2.85 in men; OR = 2.28; 95% CI: 1.57–3.31 in women), and FPG (OR = 1.96; 95% CI: 1.45–2.65 in men; OR = 1.39; 95% CI: 1.06–1.82 in women) had a higher risk for hypertension accompanied by HHcy compared to subjects with an ideal CVH metrics status.

Age was classified into four groups: 35–45, 45–55, 55–65, and >65 years. Multivariable logistic regression analyses were conducted to assess the associations between modifiable risk factors and hypertension accompanied by HHcy in the four age groups, after having adjusted for the 19 covariates as described above. Table 4 shows the ORs and 95% CIs of hypertension accompanied by HHcy. TG and LDL were both independent risk factors for hypertension accompanied by HHcy in the four age groups. According to the AHA’s criteria, BMI, physical activity, diet score, TC, and FPG were classified into three groups: ideal, intermediate, and poor. In each age group, subjects with a poor status for BMI, physical activity, TC, and FPG had a higher risk for hypertension accompanied by HHcy compared to subjects with an ideal CVH metrics status. For subjects in the 35–45 years group, a poor diet score was also a risk factor (OR = 1.97, 95% CI: 1.28–3.03) for hypertension accompanied by HHcy.

## 4. Discussion

Our study mainly found that the prevalence of hypertension accompanied by HHcy was so high that it reached 45.1% of our study population and accounted for 86.8% of the total participants with hypertension in the rural areas of northeast China. Obesity, diabetes, dyslipidemia, and inactive physical activities were modifiable risk factors of hypertension accompanied by HHcy.

A series of factors could increase the Hcy level, such as genetic factors, diet factors (lack of vitamin B12 and folic acid), and lifestyle factors (such as smoking), among which, genetic factors played a key role [14]. Methylenetetrahydrofolate reductase (MTHFR) was the crucial enzyme that affected the metabolism of Hcy, and the genetic polymorphism could upset the balance between the production and degradation of Hcy, resulting in HHcy [28,29,30]. The serum concentration of Hcy was significantly higher in the Chinese population than in western populations [30,31]. The prevalence of HHcy varied largely because the definition was different in different studies. Before 2006, HHcy was defined as the level of Hcy > 15 μmol/L in Europe and Iran [32,33]; while in 2006, HHcy was redefined as the level of Hcy > 10 μmol/L according to ASA and AHA [8,34,35]. Therefore, it was difficult to compare the prevalence of HHcy in different studies. The mean serum concentrations of Hcy were 13.56 μmol/L among Kazakh/Uyghur adults in western China [14], 20.15 μmol/L among hospitalized patients in central China [15], and 17.29 μmol/L in our study. Besides genetic factors, a lack of vitamin B12 and folic acid in the diet was another important risk factor of hypertension accompanied by HHcy [14]. The special dietary patterns in the rural population of northeast China could result in a lack of vitamin B12 and folic acid, resulting in a higher serum concentration of Hcy. Cao et al., using the same hospital as this study, found that the folic acid levels in northeast China were very low (mean value: 8.46 nmol/L), which may be another reason why the prevalence of HHcy was dramatically high in this area [36]. In the CSPPT, a randomized, double-blind, actively controlled trial demonstrated that the folic acid levels among adults with hypertension in China was 8.1 ng/mL (18.4 nmol/L) at the baseline [37]. As a previous study has suggested, the different dietary patterns between rural and urban areas may contribute to the observed differences in Hcy [14]. In our study, the mean diet score was only 2.2, suggesting that the diet pattern was poor in vegetables, but rich in fat, deep-fried, and smoked foods. During the cold winter from November to April in the second year of the study, subjects in this area lacked fresh vegetables and mainly ate meat, tofu, and pickles, such as Chinese sauerkraut. The unique climate and geographical environment in northeast China meant that the dietary patterns were similar in the rural populations, which may be the main reason why the diet scores were so low and showed no differences among the four groups in our study. Further studies are still needed in this area to detect the genotypes and the serum concentrations of vitamin B12 and folic acid when analyzing the risk factors of hypertension accompanied by HHcy.

According to a national survey conducted by the China National Survey of Chronic Kidney Disease Working Group, the adjusted prevalence of hypertension among adults aged 18 years or older was 29.6% in 2010 in China [38]. Hu et al. found that the prevalence of hypertension accompanied by HHcy was 75.0% for hypertensive patients (91% in men and 60% in women, respectively) [6]. Our study found that the prevalence of hypertension was 52.0% and hypertension accompanied by HHcy accounted for 86.8% of the total participants with hypertension, which were both higher than those in other studies. According to a national survey in 2007, more than seven million people in China suffered from a stroke, the prevalence of which was 5.4% [39]. Our previous study found that the prevalence of stroke was 8.9% in the rural population of northeast China, which was also higher than the mean level [40]. It is now well accepted that hypertension and HHcy have a synergetic effect on stroke. One perspective study based on 40,000 subjects with a six-year follow-up period found that patients with hypertension and HHcy had a 3.6-fold and 8.2-fold risk of suffering from a stroke, respectively, while patients with hypertension accompanied by HHcy had a 12.1-fold risk of suffering from a stroke [41]. Another nationwide study from the United States also confirmed that patients with a combination of HHcy and hypertension were substantially more likely to have a stroke compared to patients without either condition (OR = 12.02 in men and OR = 17.34 in women), after having adjusted for 17 covariates [42]. However, compared to previous studies on the risk factors of hypertension [43], we found that the risk factors of hypertension accompanied by HHcy and simple hypertension were almost the same, including age, gender, obesity, diabetes, dyslipidemia, and inactive physical activities. However, it was still of great significance to study hypertension accompanied by HHcy for reducing the damage to target organs, such as strokes in hypertensive patients. Our previous study found that among subjects with hypertension, 43.5% were aware of the diagnosis, and 31.2% were taking antihypertensive medications, but only 6% had their BP controlled [3]. We speculated that the high prevalence of hypertension accompanied by HHcy may be one of the reasons why the control rate of hypertension was so low. Simple antihypertensive therapy may be not so efficient in the presence of HHcy.

It is worth noting that HHcy could promote the development of hypertension [13,44], the underlying mechanism of which is too complex to be determined. However, previous studies have suggested that Hcy could inhibit endothelial cell growth, increase oxidative stress, injury endothelial repair, cause endothelial dysfunction, and promote vascular remodeling and inflammatory monocyte differentiation, which could collectively accelerate hypertension and other CVD [45,46,47,48]. The mean value of Hcy was very high (17.29 μmol/L) and the prevalence of HHcy reached 84.1% in our study population. This could partly explain why the prevalence of hypertension and stroke in this area were higher than those in other studies. As shown in Figure 2, the prevalence of simple hypertension decreased with age, suggesting that the prevalence of hypertension accompanied by HHcy dramatically increased with age. In other words, older hypertension patients also often had HHcy. Therefore, it was necessary to control the Hcy level when treating hypertension in China [41]. Indeed, the CSPPT study [37] has confirmed that after 4.5 years of treatment, the enalapril + folic acid treatment could significantly reduce the risk of a first stroke (hazard ratio = 0.79) and composite cardiovascular events consisting of cardiovascular death, myocardial infarction, and stroke (hazard ratio = 0.80) compared with only enalapril treatment. This result was further confirmed by a prospective, nested case-control study in 2015, which demonstrated that those with hypertension accompanied by HHcy could particularly benefit from Hcy-lowering therapy, along with anti-hypertension therapy, in Chinese populations [35]. Therefore, when carrying out antihypertensive therapy in the future, we need to regard the role of folic acid supplementation.

Additionally, in China, previous studies from other countries have also demonstrated that HHcy and hypertension often coexist, though they did not assess the interactive effect of HHcy and hypertension on CVD such as stroke. Towfighi A. et al. demonstrated that subjects with a combination of HHcy and hypertension were at a higher risk of suffering from a prevalent stroke compared to subjects without either condition [42]. The combination of HHcy and hypertension was equal to the concept of hypertension accompanied by HHcy in our study [42]. In the nationally representative study of 17,061 persons from the United States, the prevalence of hypertension was 41.7% and subjects with a combination of HHcy and hypertension (i.e., hypertension accompanied by HHcy) accounted for 39.4% of hypertensive patients [42]. In another study based on European populations including 25,489 subjects aged > 20 years, de Bree A. et al. found that the prevalence of HHcy in combination with hypertension was 18.7% [32]. Besides, de Bree A. et al. defined HHcy as a Hcy concentration >15 μmol/L [32], which meant that the prevalence of hypertension accompanied by HHcy would be much higher if they defined HHcy as Hcy concentration >10 μmol/L. On one hand, the concept of hypertension accompanied by HHcy is not well accepted; on the other hand, the prevalence of hypertension accompanied by HHcy is substantially high in other countries. Therefore, in future trials, it will be important to study hypertension accompanied by HHcy and its relationship with CVD outcomes in different populations besides those who are Chinese.

## 5. Limitations 

Our study had some limitations. First, this was a cross-sectional study, which can't identify a cause-effect relationship when interpreting the observed results. In the future, prospective studies are needed for further investigation of these findings. Second, our study did not analyze genotypes and the serum concentrations of vitamin B12 and folic acid. Third, the potential misclassification of exposure may have resulted due to a lack of Hcy information for ~50% of the sample.

## 6. Conclusions

In summary, our study showed that the prevalence of hypertension accompanied by HHcy was seriously high. Obesity, diabetes, dyslipidemia, and inactive physical activity were independently modifiable risk factors of hypertension accompanied by HHcy in the rural population of northeast China.

## Figures and Tables

**Table 1:** Baseline characteristics of the study population.

Variables	Control Group ^1^	Simple Hypertension Group	Simple HHcy Group	Hypertension Accompanied by HHcy Group	*p* Value
	N = 592	N = 449	N = 2545	N = 2943	
Age (year)	45.8 ± 7.8	51.5 ± 8.8	51.8 ± 10.0	59.0 ± 10.1	<0.001
Male (%)	65 (11.0)	72 (16.0)	1352 (53.1)	1501 (51.0)	<0.001
Spouse (live, %)	568 (95.9)	423 (94.2)	2373 (93.2)	2549 (86.6)	<0.001
Education (%)					<0.001
Primary school or below	198 (33.4)	230 (51.2)	1151 (45.2)	1662 (56.5)	
Middle school	341 (57.6)	180 (40.1)	1156 (45.4)	1030 (35.0)	
High school or above	53 (9.0)	39 (8.7)	238 (9.4)	251 (8.5)	
Family income (%)					<0.001
≤5000 (CNY/year)	46 (7.8)	67 (14.9)	381 (15.0)	678 (23.0)	
5000–20,000 (CNY/year)	340 (57.4)	255 (56.8)	1414 (55.6)	1690 (57.4)	
>20,000 (CNY/year)	206 (34.8)	127 (28.3)	750 (29.5)	575 (19.5)	
Race					0.022
Han	547 (92.4)	413(92.0)	2415 (94.9)	2772 (94.2)	
Others ^a^	45 (7.6)	36 (8.0)	130 (5.1)	171 (5.8)	
Current smoking (%)	106 (17.9)	108 (24.1)	1088 (42.8)	1135 (38.6)	<0.01
Current drinking (%)	50 (8.4)	56 (12.5)	615 (24.2)	730 (24.8)	<0.01
Diet score	2.2 ± 1.1	2.2 ± 1.2	2.3 ± 1.2	2.2 ± 1.2	0.059
eGFR (mL/min/1.73 m^2^)	104.3 ± 12.0	103.2 ± 13.2	94.6 ± 14.5	90.2 ± 15.9	<0.001
SBP (mmHg)	123.1 ± 10.4	157.2 ± 17.5	123.7 ± 10.0	161.0 ± 20.9	<0.001
DBP (mmHg)	74.0 ± 7.5	87.5 ± 9.9	74.6 ± 7.6	88.4 ± 11.6	<0.001
BMI (kg/m^2^)	24.6 ± 4.0	25.9 ± 3.7	23.8 ± 3.5	25.5 ± 3.8	<0.001
WC (cm)	80.2 ± 9.4	81.8 ± 10.7	82.3 ± 8.9	83.3 ± 9.4	<0.001
FPG (mmol/L)	5.58 ± 1.71	6.27 ± 2.36	5.61 ± 1.27	6.10 ± 1.92	<0.001
TC (mmol/L)	4.85 ± 1.09	5.13 ± 1.12	4.90 ± 0.95	5.28 ± 1.05	<0.001
TG (mmol/L)	1.47 ± 2.19	1.85 ± 2.44	1.46 ± 1.21	1.84 ± 1.59	<0.001
LDL (mmol/L)	2.66 ± 0.69	2.89 ± 0.81	2.67 ± 0.69	2.99 ± 0.82	<0.001
HDL (mmol/L)	1.47 ± 0.38	1.50 ± 0.41	1.41 ± 0.37	1.44 ± 0.43	<0.001
Cr (mmol/L)	59.77 ± 10.07	57.37 ± 11.76	72.27 ± 12.64	72.30 ± 29.01	<0.001
BuN(mmol/L)	5.19 ± 2.07	5.16 ± 1.42	5.68 ± 2.10	5.82± 2.08	<0.001
UA(μmol/L)	242.3 ± 65.3	249.9 ± 73.5	299.5 ± 84.7	306.8 ± 89.5	<0.001
Hcy (μmol/L)	8.33 ± 1.46	8.28 ± 1.55	17.83 ± 10.86	20.02 ± 14.00	<0.001

Data are expressed as the mean (SD) or as *n* (%). ^1^ Control group was defined as participants without hypertension and HHcy, regardless of other comorbidities. ^a^ Including some ethnic minorities in China, such as Mongol and Manchu. BMI: body mass index; BuN: blood urea nitrogen; CNY: China Yuan; Cr: Creatinine; DBP: diastolic blood pressure; eGFR: estimated glomerular filtration rate; FPG: fasting plasma glucose; Hcy: homocysteine; HHcy: high homocysteine; HDL: high-density lipoprotein cholesterol; LDL: low-density lipoprotein cholesterol; SBP: systolic blood pressure; TC: total cholesterol; TG: triglycerides; UA: uric acid; WC: waist circumference.

**Table 2:** The distribution of six CVH metrics according to BP status and Hcy concentrations.

Variables	Control Group ^1^	Simple Hypertension Group	Simple HHcy Group	Hypertension Accompanied by HHcy Group	*p* Value
	N = 592	N = 449	N = 2545	N = 2943	
**Current smoking**					<0.001
Ideal	486 (82.1)	334 (74.7)	1432 (56.3)	1778 (60.4)	
Intermediate	0 (0)	7 (1.6)	25 (1.0)	30 (1.0)	
Poor	106 (17.9)	108 (24.1)	1088 (42.7)	1135 (38.6)	
**BMI**					<0.001
Ideal	214 (36.1)	87 (19.4)	1093 (42.9)	764 (26.0)	
Intermediate	263 (44.4)	224 (49.9)	1092 (42.9)	1385 (47.0)	
Poor	115 (19.4)	138 (30.7)	360 (14.2)	794 (27.0)	
**Physical activity**					<0.001
Ideal	327 (55.2)	246 (54.8)	1496 (58.8)	1243 (42.2)	
Intermediate	109 (18.4)	96 (21.4)	504 (19.8)	569 (19.3)	
Poor	156 (26.4)	107 (23.8)	545 (21.4)	1131 (38.4)	
**Diet score**					0.205
Ideal	54 (9.1)	54 (12.0)	297 (11.7)	318 (10.8)	
Intermediate	374 (63.2)	266 (59.2)	1570 (61.7)	1762 (59.9)	
Poor	164 (27.7)	129 (28.8)	678 (26.6)	863 (29.3)	
**TC**					<0.001
Ideal	403 (68.1)	257 (57.2)	1650 (64.8)	1477 (50.2)	
Intermediate	136 (23.0)	128 (28.5)	655 (25.7)	961 (32.7)	
Poor	53 (8.9)	64 (14.3)	240 (9.4)	505 (17.2)	
**FPG**					<0.001
Ideal	419 (70.8)	210 (46.8)	1584 (62.2)	1366 (46.4)	
Intermediate	144 (24.3)	172 (38.3)	824 (32.4)	1192 (40.5)	
Poor	29 (4.9)	67 (14.9)	137 (5.4)	385 (13.1)	

Data are expressed as *n* (%). ^1^ Control group was defined as participants without hypertension and HHcy, regardless of other comorbidities. CVH: cardiovascular health; BP: blood pressure.

**Table 3:** Multivariable logistic regression analyses for associations between modifiable risk factors and hypertension accompanied by HHcy classified by gender ^a^.

Variables	OR (95% CI)	
	Men	Women
TG (per 1 mmol/L)	1.12 (1.06, 1.19)	1.09 (1.02, 1.16)
LDL(per 1 mmol/L)	1.60 (1.34, 1.90)	1.69 (1.43, 1.99)
**BMI**		
Ideal	1 (Reference)	1 (Reference)
Intermediate	2.08 (1.71, 2.52)	1.85 (1.52, 2.25)
Poor	3.41 (2.63, 4.41)	3.12 (2.45, 3.96)
**TC**		
Ideal	1 (Reference)	1 (Reference)
Intermediate	1.27 (0.92, 1.77)	1.79 (1.35, 2.39)
Poor	1.88 (1.24, 2.85)	2.28 (1.57, 3.31)
**FPG**		
Ideal	1 (Reference)	1 (Reference)
Intermediate	1.29 (1.09, 1.53)	1.11 (0.94, 1.32)
Poor	1.96 (1.45, 2.65)	1.39 (1.06, 1.82)

^a^ adjusted for age, race, marital status, education, family income, smoking, drinking, physical activity, diet score, BMI, WC, TC, TG, LDL, HDL, eGFR, UA, Cr, BuN.

**Table 4:** Multiple logistic regression analysis of modifiable risk factors and the prevalence of hypertension accompanied by HHcy by age ^a^.

Variables	OR (95% CI)			
	35–45 years	45–55 years	55–65 years	>65 years
TG (per 1 mmol/L)	1.14 (1.04, 1.26)	1.15 (1.06, 1.25)	1.13 (1.04, 1.22)	1.09 (0.99, 1.21)
LDL (per 1 mmol/L)	2.03 (1.56, 2.64)	1.66 (1.36, 2.02)	1.84 (1.48, 2.29)	1.87 (1.39, 2.50)
**BMI**				
Ideal	1 (Reference)	1 (Reference)	1 (Reference)	1 (Reference)
Intermediate	1.47 (1.10, 1.97)	1.71 (1.35, 2.16)	2.15 (1.68, 2.75)	1.85 (1.32, 2.60)
Poor	2.47 (1.71, 3.55)	2.31 (1.70, 3.13)	3.44 (2.50, 4.73)	3.16 (2.08, 4.80)
**Physical activity**				
Ideal	1 (Reference)	1 (Reference)	1 (Reference)	1 (Reference)
Intermediate	0.98 (0.72, 1.34)	1.17 (0.90, 1.51)	1.06 (0.81, 1.38)	0.81 (0.56, 1.19)
Poor	1.61 (1.20, 2.94)	1.60 (1.27, 2.02)	1.42 (1.10, 1.82)	1.58 (1.13, 2.21)
**Diet score**				
Ideal	1 (Reference)	1 (Reference)	1 (Reference)	1 (Reference)
Intermediate	1.61 (1.10, 2.36)	1.02 (0.74, 1.42)	0.78 (0.56, 1.08)	0.81 (0.51, 1.27)
Poor	1.97 (1.28, 3.03)	1.06 (0.74, 1.52)	0.77 (0.53, 1.11)	0.83 (0.50, 1.38)
**TC**				
Ideal	1 (Reference)	1 (Reference)	1 (Reference)	1 (Reference)
Intermediate	1.58 (0.99, 2.51)	1.77 (1.23, 2.56)	1.56 (1.06, 2.31)	1.96 (1.14, 3.37)
Poor	2.56 (1.43, 4.61)	2.04 (1.27, 3.27)	2.28 (1.37, 3.80)	2.56 (1.28, 5.12)
**FPG**				
Ideal	1 (Reference)	1 (Reference)	1 (Reference)	1 (Reference)
Intermediate	1.44 (1.12, 1.86)	1.37 (1.11, 1.70)	1.04 (0.84, 1.30)	1.38 (1.03, 1.86)
Poor	1.88 (1.20, 2.94)	1.97 (1.42, 2.75)	1.76 (1.22, 2.53)	1.51 (0.88, 2.58)

^a^ adjusted for gender, race, marital status, education, family income, smoking, drinking, physical activity, diet score, BMI, WC, TC, TG, LDL, HDL, eGFR, UA, Cr, BuN.
